# Case report: Severe asymptomatic hypertriglyceridemia associated with long-term low-dose rapamycin administration in a healthy middle-aged Labrador retriever

**DOI:** 10.3389/fvets.2023.1285498

**Published:** 2023-11-29

**Authors:** Jeremy B. Evans, Lucy Chou, Matt Kaeberlein, Daniel E.L. Promislow, Kate E. Creevy

**Affiliations:** ^1^Department of Small Animal Clinical Sciences, Texas A&M School of Veterinary Medicine & Biomedical Sciences, College Station, TX, United States; ^2^Department of Laboratory Medicine and Pathology, University of Washington School of Medicine, Seattle, WA, United States; ^3^Optispan, Inc., Seattle, WA, United States; ^4^Department of Biology, University of Washington, Seattle, WA, United States

**Keywords:** dog, lipemia, dyslipidemia, side effect, adverse effect

## Abstract

Rapamycin is an mTOR inhibitor that has been shown to extend the lifespan of laboratory model organisms. In humans, rapamycin is used at higher doses as an immunosuppressive medication to prevent organ rejection. Numerous adverse effects are seen with rapamycin treatment in humans, with one of the most common being dysregulation of lipid metabolism. In humans, this often manifests as mild to moderate serum lipid elevations, with a small subset developing extreme triglyceride elevations. This case report describes an eight-year-old, castrated male, clinically healthy Labrador retriever who developed severe hypertriglyceridemia associated with low-dose rapamycin administration over a six-month period. During this time, the dog was asymptomatic and displayed no other clinical abnormalities, aside from a progressive lipemia. Within 15 days of discontinuing rapamycin treatment, and with no targeted lipemic intervention, the dog’s lipemia and hypertriglyceridemia completely resolved.

## Introduction

Rapamycin (also known as sirolimus), an antifungal macrolide, is an inhibitor of the mammalian target of rapamycin (mTOR), a serine/threonine protein kinase responsible for regulating cell growth, proliferation, and lifespan by integrating nutrient availability, cell signaling, and stressors (1–3). Rapamycin has displayed promising anti-tumoral activity against various types of neoplasia (1, 3–8), and due to its immunosuppressive effects, it is often utilized to prevent acute rejection in human renal transplant recipients (9–14). Additionally, one of the more remarkable discoveries regarding rapamycin is its ability to significantly extend lifespan in multiple laboratory animal models (15–26). Though the effect of rapamycin on canine longevity has not yet been assessed in dogs, previous studies have demonstrated safe administration protocols which resulted in improved cardiac function and promise in treating canine glycogen storage disease (27–29).

In humans and rodents, rapamycin is associated with various systemic side effects and clinicopathologic changes (30–35). Among these, hypercholesterolemia and hypertriglyceridemia are some of the more commonly observed clinicopathologic changes (30, 31, 36–38). The exact mechanism for dyslipidemias is not precisely understood, but may be related to the inhibition of insulin-mediated lipid metabolism, decreased lipoprotein lipase activity, and/or an increase in circulating lipoproteins (38–41).While the vast majority of blood lipid elevations are mild to moderate, there are rare reports in human literature describing severe triglyceride elevations developing secondary to rapamycin therapy (42–44). In the limited studies involving dogs, low-dose rapamycin administration has not been associated with any significant side effects other than erythrocyte microcytosis and thrombocytopenia (27, 28).

## Case description

A healthy eight-year-old male neutered Labrador retriever was recruited into a study of the effects of long-term, low-dose intermittent rapamycin administration in companion dogs. This trial and its results have been reported (45) and will be summarized briefly here. Conventional media, websites and targeted local community outreach in the Bryan/College Station, Texas area were used to solicit enrollment between May 2018 and February 2019. The enrollment process consisted of evaluation at three stages: owner survey responses, review of the dog’s veterinary medical records, and physical examination/diagnostic findings at an in-hospital appointment at the Texas A&M University Veterinary Medical Teaching Hospital (TAMU VMTH). Participants for whom all responses and findings met the inclusion criteria were enrolled. In-hospital follow-up examinations were scheduled at the three-, six-, and 12-month study time points at TAMU VMTH. In between hospital visits, owners were asked to complete surveys about their dogs’ condition every two weeks online. At the baseline screening examination, dogs were evaluated by a board-certified small animal internist and cardiologist for medical history and physical examination abnormalities or signs suggestive of underlying systemic illness. Diagnostics were performed by the TAMU VMTH Clinical Pathology Laboratory, Clinical Immunology Laboratory, and Gastrointestinal Laboratory and included a heartworm antigen test, complete blood count, biochemistry panel, total thyroxine (TT4) concentration (if TT4 was low, reflex free thyroxine [free T4] and thyroid stimulating hormone [TSH] tests were performed), Chagas disease (*Trypanosoma cruzi*) serology, and urinalysis. Blood pressure (indirect systolic blood pressure measured using Doppler), electrocardiogram, and echocardiogram were performed by small animal internal medicine and cardiology faculty and veterinary technicians. Serum was banked from each dog at each visit by flash-freezing in liquid nitrogen and storing at -80°C. During the follow-up examinations, most of the same diagnostics were performed, except for heartworm disease, Chagas disease, and thyroid testing. All procedures for this study were reviewed and approved by the TAMU Institutional Animal Care and Use Committee (IACUC 2017–0125).

Seventeen dogs were enrolled into the one-year clinical trial. The randomized, placebo-controlled, double-masked trial included six months of treatment (rapamycin or placebo) and six additional months of monitoring. Owners were instructed to administer study medication to their dogs three times per week on Monday, Wednesday, and Friday in the morning. Dogs in the rapamycin group were administered a 0.025 mg/kg dose by mouth on administration days. After randomization, nine dogs were placed into the rapamycin group and eight were placed into the placebo group; the participant described in this report was placed into the rapamycin treatment group. Labwork in all other participants was largely unremarkable throughout the trial (45). One other rapamycin-treated dog had a positive lipemia index reported by the clinical pathology laboratory at the three-and six-month rechecks that was not further pursued.

The participant of this report had a medical history of dermatologic issues primarily consisting of ear infections and dermatitis. Associated clinical signs (mainly, pruritus) were historically well-controlled with oclacitinib (Apoquel, Zoetis US, Parsippany, NJ). Per the owner, there were no significant events in the participant’s medical history since being obtained as a puppy, and the only prior surgery was an elective castration.

During the initial screening physical exam, the only abnormality appreciated in this participant was over-conditioning (body condition score = 6/9). The previously outlined screening diagnostics were performed, and no significant abnormalities or evidence of systemic disease were identified. For the purposes of this report, presentation of serial diagnostic results will focus on cholesterol concentration, triglyceride concentration, and gross lipemia, as all other diagnostic monitoring parameters remained normal throughout the duration of the study. Baseline results included a normal cholesterol concentration (247 mg/dL, reference interval 120–247 mg/dL), and lack of gross lipemia (qualitatively scored lipemia index = 0) on blood samples. Triglyceride concentration was not included on the hospital’s or the study’s routine biochemistry panel. Total thyroxine concentration was 2.37 mcg/dL (reference interval, 1.7–3.6).

At the three-month evaluation, the patient displayed no clinical abnormalities, and diagnostic monitoring parameters remained unremarkable with normal cholesterol (243 mg/dL), and overt lipemia with a lipemia index of 3+ on a blood sample that was believed to have been fasted. The finding of lipemia was discussed with the owner and it was recommended to obtain a second sample to directly measure triglycerides. However, it was then discovered that the dog had likely eaten a fatty treat on the morning of the appointment, which seemed a plausible explanation for the visually detected lipemia. Additional testing was not pursued at this time.

**Figure 1:**
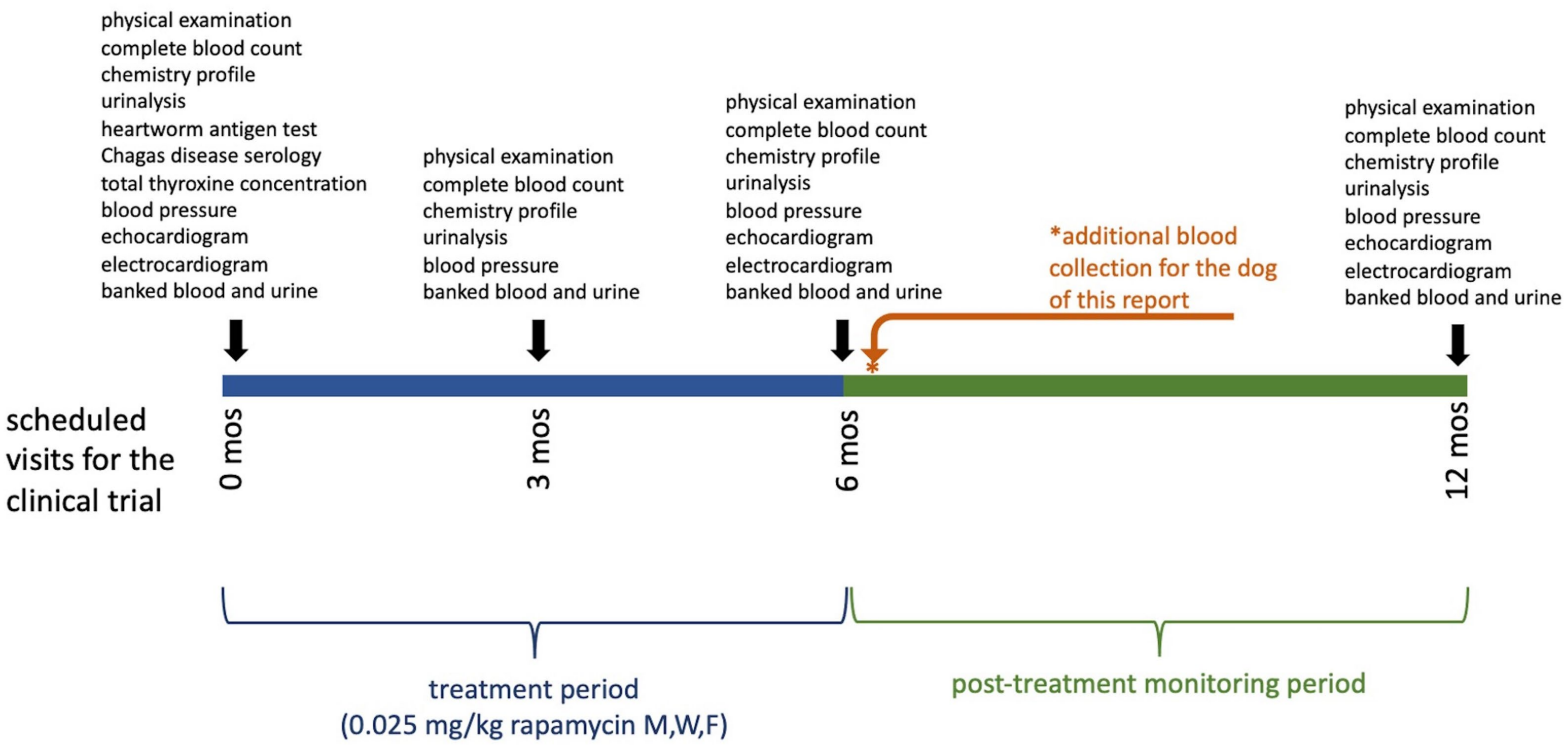
Clinical trial timeline displaying the treatment period and sampling intervals for dogs in this study. Examinations performed and samples collected at each time point are shown above the bar. The dog of this report had an additional sampling timepoint after recognition of the severe hypertriglyceridemia, indicated by the asterisk (*).

In this study, the treatment period was six months, with six additional months of follow-up. This participant received his final dose of study medication eight days before his six-month examination. At the time of the examination, the participant displayed no clinical abnormalities, and diagnostic monitoring parameters remained generally unremarkable including normal cholesterol (232 mg/dL). However, gross lipemia was noted again (lipemia index 4+) and the participant was confirmed to have been fasted prior to the appointment. This fasting gross lipemia prompted measurement of triglyceride concentration. Triglyceride concentration was severely elevated at 2167 mg/dL (reference interval, 11–140 mg/dL). This value was confirmed by repeat analysis and serial dilution as part of the standard operating procedure of the laboratory. Because of the hypertriglyceridemia, the treatment assignment was unmasked to a board-certified internist not associated with the study so that the dog’s clinical condition could be monitored; the treatment assignment was revealed to have been rapamycin. Follow-up biochemistry was recommended to be performed in one week and was submitted to a veterinary reference laboratory by the owner’s local primary care veterinarian. At this time (i.e., 15 days after the final dose of rapamycin) triglyceride concentration was normal (87 mg/dL, reference interval, 29–291 mg/dL). TT4 (which had been submitted to rule out development of hypothyroidism as a cause of the triglyceride elevation) was also normal (1.1 mcg/dL, reference interval, 0.8–3.5 mcg/dL), and all other results from the complete blood count, biochemistry profile and urinalysis were also normal. Figure 1 displays the timeline of the study, the treatment period, and the sampling intervals.

**Figure 2:**
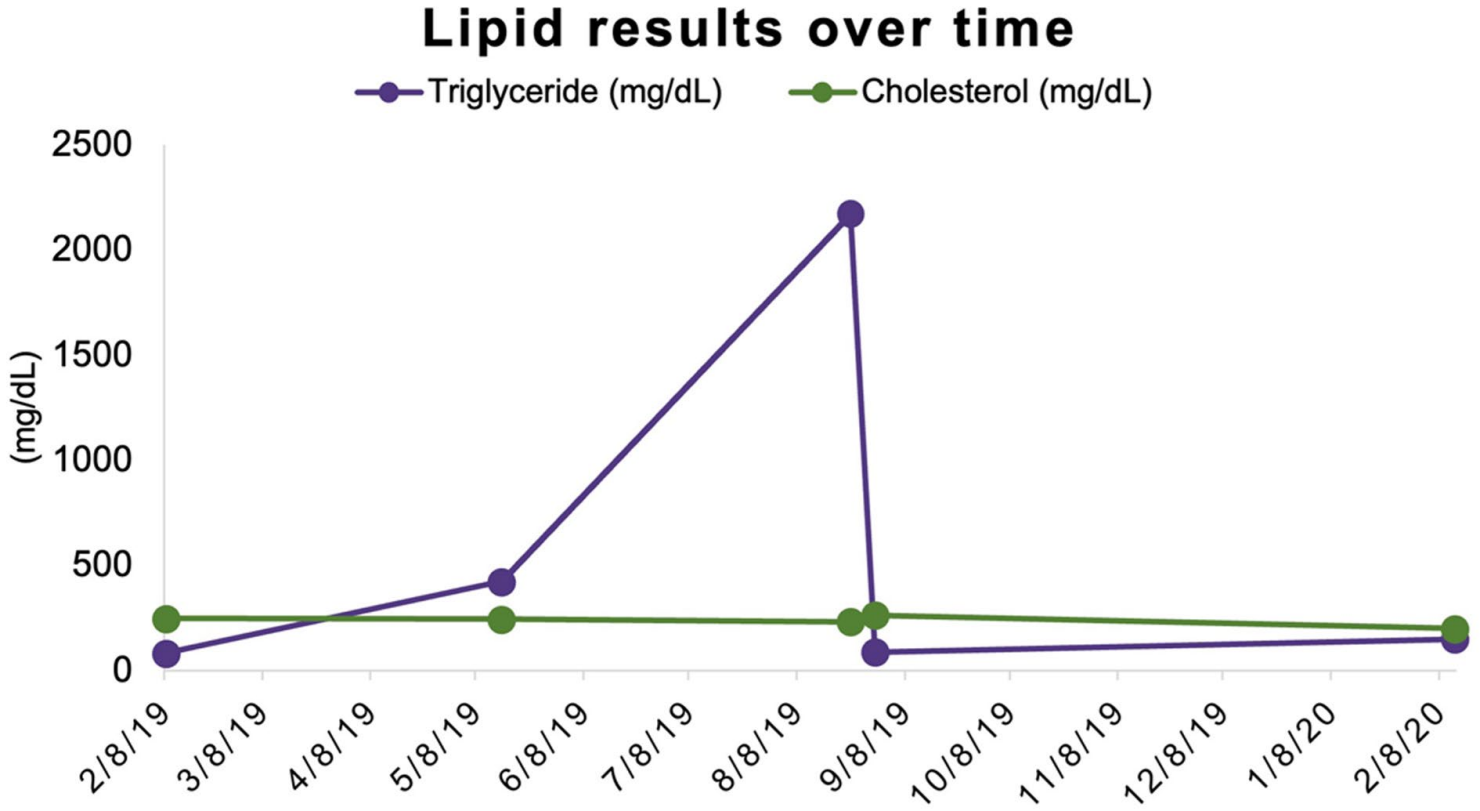
Triglyceride and cholesterol concentrations over time. Triglyceride and cholesterol concentrations share a scale (mg/dL) on the *y*-axis.

Banked serum samples from this participant’s baseline and three-month evaluations were used to retrospectively measure triglyceride concentrations and revealed results of 82 mg/dL and 422 mg/dL, respectively. Triglyceride concentration was assessed once more at the time of the participant’s final study-related examination to TAMU VMTH at 12 months (6 months after completion of study medication administration) and was 149 mg/dL (reference interval 11–140 mg/dL) (Table 1; Figure 2).

**Table 1:** Reported values of this participant’s triglyceride concentration, cholesterol concentration, lipemia index, and total thyroxine (TT4) concentration at the baseline examination (2/8/19) and subsequently.

Date	Triglyceride (mg/dL)	Cholesterol (mg/dL)	Lipemia (+)	T4 (mcg/dL)
2/8/19	82	247	0+	2.37
5/15/19	422	243	3+	–
8/23/19	2,167	232	4+	–
8/30/19	87	262	–	1.1
2/12/20	149	199	1+	–

## Discussion

This case report represents the first description of a healthy, middle-aged, privately owned dog developing a severe hypertriglyceridemia while receiving low-dose rapamycin. Though it is difficult to establish direct causation, the rigorous screening process to rule-out the presence of systemic disease, the severe and asymptomatic nature of the hypertriglyceridemia, and the complete resolution of the hypertriglyceridemia within 15 days of discontinuing rapamycin administration is highly suggestive that rapamycin was the cause of the hypertriglyceridemia. Hypertriglyceridemia can be secondary to multiple causes in the dog and a variety of alternate explanations for this dog’s hypertriglyceridemia were considered. The most common causes of hypertriglyceridemia in dogs are hypothyroidism or recent food ingestion, although even in these circumstances, it is highly unusual for the increase to be as extreme as the value observed in this case (46). In this dog, a euthyroid status was confirmed both at the beginning of the study as well as shortly after the six-month examination at which hypertriglyceridemia had been documented so hypothyroidism cannot explain his hypertriglyceridemia. At the time of the six-month examination finding of hypertriglyceridemia and the biochemistry recheck seven days later, the owner confirmed that no recent meal had been provided. It is possible that the owner was mistaken about this and that the dog had inadvertently been fed by another member of the household prior to blood collection; even so, the magnitude of the hypertriglyceridemia would be unusually severe for a post-prandial increase. Additionally, the dog was fed a commercial, dry, adult-formula dog food, with no changes in diet throughout the trial, so a difference in principal dietary components does not explain the change. Primary hypertriglyceridemia also occurs in dogs, notably Miniature Schnauzers, but given the complete resolution of the lipemia without targeted intervention other than discontinuation of rapamycin, primary hypertriglyceridemia was not present in this case. Other endocrinopathies, pancreatitis, protein-losing nephropathy and cholestasis are additional causes of hypertriglyceridemia in dogs that were not present in this case (47). This dog had a normal physical examination, and normal CBC, chemistry profile and urinalysis (other than hypertriglyceridemia) at each examination. Diabetes mellitus is ruled out by normal blood glucose and lack of glycosuria. While a low-dose dexamethasone test was not performed, the absence of polyuria, polydipsia, endocrine alopecia, increase in alkaline phosphatase activity, stress leukogram, thrombocytosis, or dilute urine concentration makes hyperadrenocorticism implausible. The dog had no clinical signs of pancreatitis such as vomiting, diarrhea, inappetence or abdominal pain at any time. Urine protein: creatinine was not performed, however, in the setting of well-concentrated urine (1.039–1.056) at each visit, the dog had only trace dipstick proteinuria at any time, and had a consistently normal serum albumin, thus protein-losing nephropathy does not explain the hypertriglyceridemia. Finally, the dog had no increase in serum cholesterol, total bilirubin, or liver enzymes (alanine aminotransferase, alkaline phosphatase, gamma glutamyltransferase) at any time to support a diagnosis of cholestasis. Given those findings and the rapid complete resolution of the hypertriglyceridemia with no intervention other than rapamycin discontinuation, no alternate explanation for this dog’s severe hypertriglyceridemia was found.

In human patients treated with rapamycin and its derivative mTOR inhibitor, everolimus, moderate hypertriglyceridemia and mild to moderate hypercholesterolemia are relatively common while severe hypertriglyceridemia is rare (42, 48–51). At this time, there is no clear consensus whether the development of dyslipidemia associated with rapamycin administration in people is most influenced by the dose, the serum concentration, or the duration of therapy (42, 52). The precise mechanisms of these dyslipidemias are uncertain, but likely result from alterations to insulin signaling caused by mTOR inhibition that subsequently inhibit the activity of lipases on circulating lipoproteins, and/or alterations in degradation of apolipoprotein B100 (51, 53, 54). It is possible that while mild to moderate hyperlipidemia is dose-dependent, severe hyperlipidemia is dose-independent and related to other patient factors. There is some evidence that pre-treatment lipid values can predict the presence, but not necessarily the severity, of hyperlipidemia associated with mTOR-inhibitor therapy, which suggests that individual factors (genetic, co-morbid, or otherwise) may contribute to the risk of severe increases (55). Relevant to the possibility of a genetic risk, the subject of this case report was a Labrador retriever. There were four other Labrador retrievers enrolled in the clinical trial (two in the rapamycin group), none of whom developed hyperlipidemia; detailed genetic analysis was not performed. The results of our study support the dose-independent nature of severe hyperlipidemia, as our participant was receiving a very low dose of rapamycin only three days a week. While lower doses have been used in dogs (27), those were daily intramuscular injections, and at this time our study represents the lowest reported oral rapamycin dosing schedule for dogs.

When considering the protocol used in this case and that no dog has been previously reported to develop severe dyslipidemia despite receiving equal or higher doses, this supports the unpredictable and dose-independent nature of this side effect. In humans, rapamycin is commonly used at a higher relative dose and administered daily (30, 31). This is distinctly different from our case, in which the Monday–Wednesday–Friday dosing schedule was more similar to ‘intermittent dosing’, as pharmacokinetic data obtained from other study participants revealed no detectable rapamycin in the blood 48 h post-administration (unpublished data). Additionally, unpublished data related to this study shows peak serum rapamycin concentrations were approximately 1 to 2 ng/mL, which is significantly lower than the therapeutic concentration targeted in humans (12).

In humans who develop severe hypertriglyceridemia secondary to rapamycin, it can be observed as soon as one to three months after starting the medication but might take as long as two years to develop (42, 44). In our case, the gross lipemia observed at the participant’s six-month recheck prompted the triglyceride measurement. However, when previous banked samples were retrospectively analyzed, the patient’s triglycerides were already moderately elevated at three months. The relationship to the possibility that the three-month sample was not fasted cannot be determined. Since the monitoring and banking protocol did not include blood sampling between the baseline and three-month time points, we were unable to determine when the triglyceride increase started. Still, within seven days of having serum triglycerides greater than <2000 mg/dL, and fifteen days of discontinuation of rapamycin administration, the participant’s lipemia and hypertriglyceridemia had completely resolved. A similar dramatic reduction following rapamycin discontinuation has been observed in humans, with significant improvements seen within five to ten days, and complete resolution observed as early as one month (42, 44). In humans, these triglyceride reductions are often due to a combination of rapamycin discontinuation and targeted lipemic intervention.

In dogs, the most common clinical finding associated with hypertriglyceridemia, as cause or consequence, is pancreatitis (47, 56). In contrast to humans who present with similar triglyceride concentrations, this dog was considered asymptomatic for his dyslipidemia. It is possible that some of the signs present in humans (migraines or a tight chest, for example), which are generally recognized due to self-reporting, might have gone unnoticed in our case (43, 44).

At this time, it is unknown whether repeated exposure to rapamycin would again elicit a significant increase in triglyceride concentration in this dog, as seen in humans (38). As the study participant is a privately owned dog who does not require rapamycin therapy for any reason, and hypertriglyceridemia is not without risk, it would be unethical to expose the dog to this risk in order to answer this question. Since this side effect has now been observed across multiple species, future studies are warranted to better understand how commonly hyperlipidemia is observed during rapamycin administration in dogs and other species, and to identify what factors may predispose certain individuals to this side effect.

## Data availability statement

The raw data supporting the conclusions of this article will be made available by the authors, without undue reservation.

## Ethics statement

The animal studies were approved by the Texas A&M University School of Veterinary Medicine and Biomedical Sciences Institutional Animal Care and Use Committee (IACUC) and Clinical Research Review Committee protocol number 2017-0125. Because the study involved client-owned animals, the TAMU IRB was consulted and the study was determined not to be human subjects research. Written informed consent was obtained from the owners for the participation of their animals in this study. The studies were conducted in accordance with the local legislation and institutional requirements.

## Author contributions

JE: Investigation, Writing – original draft, Writing – review & editing. LC: Investigation, Writing – original draft, Writing – review & editing. MK: Conceptualization, Funding acquisition, Writing – review & editing. DP: Conceptualization, Funding acquisition, Writing – review & editing. KC: Conceptualization, Funding acquisition, Investigation, Supervision, Writing – review & editing.
